# Hand preference, performance abilities, and hand selection in children

**DOI:** 10.3389/fpsyg.2014.00082

**Published:** 2014-02-18

**Authors:** Sara M. Scharoun, Pamela J. Bryden

**Affiliations:** ^1^Department of Kinesiology, University of WaterlooWaterloo, ON, Canada; ^2^Department of Kinesiology and Physical Education, Wilfrid Laurier UniversityWaterloo, ON, Canada

**Keywords:** handedness, preference, performance, hand selection, reaching

## Abstract

It is widely know that the pattern of human handedness is such that approximately 90% of the population is right handed with the remainder being left handed, at least in the adult population. What is less well understood is how handedness develops and at what age adult-like handedness patterns emerge. Quantified in terms of both preference and performance, a plethora of different behavioral assessments are currently in use with both children and adults. Handedness questionnaires are commonly used; however, these possess inherent limitations, considering their subjective nature. Hand performance measures have also been implemented; however, such tasks appear to measure different components of handedness. In addition to these traditional measures, handedness has been successfully assessed through observation of hand selection in reaching, which has proven to be a unique and effective manner in understanding the development of handedness in children. Research over the past several decades has demonstrated that young children display weak, inconsistent hand preference tendencies and are slower with both hands. Performance differences between the hands are larger for young children, and consistency improves with age. However, there remains some controversy surrounding the age at which hand preference and hand performance abilities can be considered fully developed. The following paper will provide a review of the literature pertaining to hand preference, performance abilities and hand selection in children in an attempt to ascertain the age at which adult-like patterns of hand preference and performance emerge.

Handedness is quite possibly the most studied human asymmetry; therefore much attention has been devoted to its assessment (e.g., Bryden, 1982; Steenhuis and Bryden, 1987, 1988, 1989). Control of the hands is contralateral, such that the right hand is under left hemisphere control and the left hand is under right hemisphere control (e.g., Annett, 1981a,b). This can be traced back to the work of Paul Broca, a nineteenth century neurologist, who hypothesized an association between neural control for language and an individual’s hand preference. Clinical observations of language impairment caused by left hemisphere insult, in combination with the knowledge of his patient’s right-handed preferences (Harris, 1993; Provins, 1997), led Broca to suggest that neural control for language mirrored an individual’s hand preference.

In most cases, the left hemisphere is responsible for language function and manual preference (Sperry, 1974; Khedr et al., 2002; Vogel et al., 2003; Forrester et al., 2013). In particular, language is lateralized in the left hemisphere in 87–96% of the human population; however, not all people are right-handed (Annett, 1981a,b; Khedr et al., 2002), as one might assume. Although most right handers do fall within this distinction, as do 60–73% of left handers, right-hemisphere control for language or bilateral distribution across the two hemispheres can be observed in a small minority of individuals. This division has been confirmed through functional transcranial sonography, a non-invasive neural imaging technique that assesses the rate of cerebral blood flow during language tasks (Knecht et al., 2000a,b). Other researchers have demonstrated similar results using repetitive transcranial magnetic stimulation (rTMS; e.g., Khedr et al., 2002) and functional magnetic resonance imaging (fMRI; e.g., Pujol et al., 1999). The association between human hand preference and language function remains a topic of debate (Vauclair, 2004).

Considering the relationship between hand preference and language lateralization, it has been suggested that right hand dominance is a uniquely human trait (Annett, 2002; McManus, 2002). It is well known that 90% of the human population is right-handed, where this proportion has remained relatively consistent for approximately 5000 years (Coren and Porac, 1977). Handedness is typically described as the hand one prefers to use for unimanual tasks (Annett, 1970a). Two distinct components include *direction *and *degree*. Direction simply quantifies whether an individual is left- or right-handed. In comparison, degree identifies how strongly a person prefers one hand to the other (Steenhuis and Bryden, 1989). It is well-known that left handers generally display less functional asymmetry than right handers (e.g., Springer and Deutsch, 1998; Yahagi and Kasai, 1999), therefore the degree to which they use their preferred hand is significantly less in comparison to right handers.

Handedness is further divided into measures of *preference* and *performance*. Hand preference identifies the preferred hand for completing a task, whereas performance differentiates between the abilities of the left and right hand on a particular task (McManus and Bryden, 1992). A relationship is commonly observed between these two constructs, such that performance abilities (i.e., skill) increases with the preferred hand (Annett, 1970b). But, this is not always the case (Jäncke et al., 1998). Research has revealed that right handers display more activation in the right hemisphere when using the left hand, than in the left hemisphere when using the right hand. It has been suggested that, in order for right handers to perform with their non-preferred, left hand, more effort is required (Jäncke et al., 1998).

Human hand preference emerges very early in an infant’s life, where genetics and environmental influences are believed to play a key role in development. Some researchers have suggested hand preference in adulthood may be predicted from lateralized motor behavior in early gestation, comparing ultrasound observation of thumb sucking (Hepper et al., 1991), and neonate palmar grasp reflex strength (Tan and Tan, 1999). It has also been suggested that infant postural preferences can guide the development of handedness (Coryell and Michel, 1978; Michel, 1981), where observations of hand preference for reaching (Marschik et al., 2008) and grasping objects (Michel et al., 2002, 2006) have been observed to parallel hand-use distributions later in life. Research has indicated that hand preference can be reliably detected from 6-months onward (see Butterworth and Hopkins, 1993 for review of handedness in infants). Both cross-sectional (Gesell and Ames, 1947; Hawn and Harris, 1983; Peters, 1983; Michel et al., 1985; Cornwell et al., 1991; Morange and Bloch, 1996; Fagard, 1998) and longitudinal studies (Coryell and Michel, 1978; Ramsay et al., 1979; Carlson and Harris, 1985; Ramsay, 1985; Michel and Harkins, 1986; McCormick and Maurer, 1988; see Michel, 1984; Provins, 1992, for reviews) with infants indicate that some degree of hand preference is evident with the emergence of voluntary grasping. Together, these findings suggest that human hand preference may manifest itself very early in life. Nevertheless, variable hand use preferences (i.e., shifting from right to left hand use) have been noted in infancy, confirming that the development of hand preference during infancy is highly malleable (Corbetta et al., 2006) and different patterns of development exist (Michel et al., 2006).

Observing hand preference from early childhood to adolescence (i.e., ages 3–12) no general consensus exists surrounding the age at which adult-like handedness is actually attained. Some researchers (Archer et al., 1988; Longoni and Orsini, 1988; McManus et al., 1988) suggest that *direction* of hand preference is fixed at age 3, further explaining that degree increases between the ages of 3–7 and more gradually until the age of 9. Based on this idea, an individual’s hand preference cannot be reliably assessed until 4 years of age (McManus, 2002). Other researchers have noted that children 3–4 years of age do not reliably select the preferred hand to perform unimanual tasks, and that it is not until the age of 6 that a clear preference can be observed (e.g., Bryden et al., 2000a). The equivocal findings here may be due to the different ways of quantifying hand preference and performance abilities in the research. Regardless, consensus has not yet been reached on the developmental milestones of handedness, and how adult-like patterns emerge. The following review will provide a synopsis of hand preference, performance abilities, and hand selection over the course of development, from early childhood to adulthood in an attempt to ascertain the age at which adult-like patterns of hand preference and unimanual skill emerge. More specifically, the first section will provide a review of traditional assessments hand preference (e.g., questionnaires), performance (e.g., pegboards, tapping tasks, etc) and performance-based measures of preference (i.e., observational assessments). The second section will explain non-traditional assessments of hand preference, paying particular attention to manual midline crossing and reaching into hemispace, as we argue that handedness assessed through the observation of hand selection in reaching provides some of the richest data regarding the development of hand preference and unimanual skill. The review will focus on development from early childhood (3-year-olds) to adolescence (12-years-old); however, some infant and adult literature will be included when necessary. As the majority of research is cross-sectional in nature, unless otherwise stated the review will primarily focus on cross-sectional data.

## DEVELOPMENT OF HANDEDNESS: TRADITIONAL ASSESSMENTS

### HAND PREFERENCE

Hand preference is typically assessed with a handedness questionnaire (McManus and Bryden, 1992). A plethora of instruments are currently in circulation, including the Crovitz–Zener Scale (Crovitz and Zener, 1962), the Annett Handedness Questionnaire (Annett, 1970a), the Edinburgh Handedness Inventory (Oldfield, 1971), the Lateral Dominance Examination (Reitan and Davidson, 1974), the Waterloo Handedness Questionnaire (WHQ; Steenhuis et al., 1990), and the Lateral Preference Inventory (Coren, 1993). Each of these questionnaires can be used to assess the direction of handedness (left- or right-handed). In addition, some enable the degree of handedness to be determined. Observing the current state of the literature, the Annett Handedness Questionnaire (Annett, 1970a), Edinburgh Handedness Inventory (Oldfield, 1971), and Waterloo Handedness Questionnaire (Steenhuis et al., 1990) are the most commonly used assessments. None of these questionnaires were explicitly designed for use with children; therefore, measuring handedness in children with questionnaires poses unique challenges, considering the inherent verbal requirements, and inability to assess children’s familiarity of specific items and tasks. Nevertheless researchers have overcome these obstacles in a variety of ways. This includes parent or teacher report, oral administration, and/or asking children to perform each of the items, while the experimenter records responses. Use of questionnaires with children is prevalent in the literature. 

In the largest survey study to date, Carrothers (1947) investigated the handedness of 225,000 school children (grades 1–12) in Michigan. The authors implemented a cross-sectional study design, observing a general tendency for left-handedness to decline with age. In order to deal with the limitations outlined previously, Carrothers (1947) relied on classroom teacher’s report of their student’s hand preference. In light of the fact that different teachers were assessing their own students, results must be interpreted with caution, due to bias from inter-rater reliability. Since Carrothers’ ( 1947) study, numerous researchers have used handedness questionnaires to assess developmental trends in hand preference. For example, Porac et al. (1980) assessed age-related changes in lateral preference (hand, eye, foot, and ear preference) in a large sample (*N* = 1964) of 8- to 100-year-olds using a 13-item behavioral-validated self-report battery. The authors did not indicate whether different procedures were implemented for younger participants. Within the battery, four questions were designed to quantify hand preference, where the remaining questions addressed foot, eye, and ear preference. Results indicated that the number of individuals classified as right-handed increases with age, where two developmental hypotheses were presented to explain findings. First, the authors noted environmental pressures toward right-handedness, highlighting that, prior to 1930, use of the left hand for writing was frowned upon (Blau, 1946). Nevertheless, their review of 34 studies from 1913 to 1976 indicated that social constraints account minimally for changes in hand preference (Porac et al., 1980). Left-handedness has become more culturally accepted in the western world; however, the number of left handers remains considerably lower in eastern cultures due to social constraints continuing to limit left-hand use (Ida and Bryden, 1996; Mandal, 1999). The authors also discuss developmental maturational processes, indicating that neural development continuing into the third decade of life (Yakovlev and Lecours, 1967) may influence the development of hand preference. 

Some researchers have asked children to perform each action in order to observe the preference for an item listed on the questionnaire. Kilshaw and Annett (1983) observed hand selection to complete the 12-items of the Annett (1970a) Handedness Questionnaire These actions included: writing, throwing a ball, holding a tennis racket, striking a match, cutting with scissors, threading a needle, sweeping with a long-handles broom, shoveling with a long-handled shovel, dealing playing cards, hammering, using a toothbrush and unscrewing the lid of a jar. The distribution of hand preference did not change as a function of age; however, younger children were notably more variable in performance than older children. Brito and Santos-Morales (1999); Brito et al. (1992) also used this method to assess the hand preference of 4- to 7-year-old (Brito et al., 1992) and 8- to 15-year-old (Brito and Santos-Morales, 1999) Brazilian children using the Edinburgh Handedness Inventory. Based on questionnaire responses, children were divided into categories of hand preference adapted from Annett (1970b) by sex and age. The distribution of laterality quotients was *J*-shaped, where the frequency of left-handedness was greater in male children as compared to female children. This *J*-shaped distribution parallels distribution of hand preference observed in adults. 

Others have opted to read questionnaire items out loud and have the experimenter record hand preference responses. This has proven successful for pre-school children as young as 2-years-old (e.g., Cavill and Bryden, 2003). Cavill and Bryden (2003) assessed handedness in 2- to 24-year-olds using the Revised WHQ (20-item). In comparison to Carrothers (1947), preference for the right hand was revealed across all age groups, where no differences among the age groups were revealed (Cavill and Bryden, 2003). These results parallel related reports in the literature (e.g., Hardyck et al., 1976; Kilshaw and Annett, 1983; Whittington and Richards, 1987; Bryden et al., 1991) which have also been unable to identify a significant change in the direction of hand preference as a function of age. That said, De Agostini et al. (1992) observed children below the age of 3 years demonstrate a significantly smaller right hand preference than noted in adults. 

More recent investigations with the WHQ have revealed that most right handers score approximately the same on questionnaire items. Regardless of age, right handers typically report a right hand preference for items on the WHQ. In comparison, left-handed children display weaker hand preference tendencies at younger ages; therefore they do not display consistent hand preference tendencies over the course of development. Young left-handers (up to 8 year s of age) report they would use their left, right, or both hands equally for items on the WHQ. As left handers approach adulthood, the number of left hand responses increases; however, left handers, as a group are less consistent in hand preference tendencies than their right-handed counterparts (e.g., Bryden et al., 2000b; Cavill and Bryden, 2003). 

Unfortunately, numerous problems exist with relying solely on self-report inventories of handedness. As mentioned previously, a number of handedness questionnaires are in circulation. As might be expected, the choice of questionnaire will undoubtedly influence results (Williams, 1991; Peters, 1998), as each possesses a unique type and number of items, and classification system. Different patterns of results can emerge solely on how handedness is classified (e.g., Peters, 1998; Steenhuis and Bryden, 1999; Bryden et al., 2005). Other concerns include researchers who select questionnaire items based on research needs (Brown et al., 2006). According to Peters (1998), researchers must “use different classification schemes, and examine how well these relate to the specific variables thought to relate to handedness and why” (p. 93). Finally, questionnaires are limited due to the inherent subjectivity, which makes administration to children and other special populations quite difficult, although clearly possible. Despite such problems a high degree of concordance between questionnaire items and observed preference in performance has been noted. Steenhuis and Bryden (1989) have observed that performance measures are related to preference items that assess the same activity, where Reib et al. (1998) have reported a 95.4% agreement. However, in young children ages 3- to 5-years of age, Bryden et al. (2007a) showed low correspondence between scores on a hand preference questionnaire and scores on an observational method of assessing hand preference (WatHand Cabinet Test described later), which includes similar items to the questionnaire. By age 6, a high degree of correspondence was found between these two measures (*r* = 0.767, *p* < 0.01). 

Summarizing the research assessing hand preference in children, research using questionnaires have revealed that right handers typically report consistent right hand preferences from early childhood to adulthood. In comparison, left handers demonstrate weak hand preference tendencies that increase as a function of age, but rarely do left handers exhibit as strong preferred hand tendencies as their right-handed counterparts. It appears that direction of hand preference may be established at a relatively young age, as suggested by several researchers (Archer et al., 1988; Longoni and Orsini, 1988; McManus et al., 1988). However, it is clear that the degree or strength of hand preference requires further refinement over the next several years. The establishment of a consistent and reliable degree of hand preference may be due to increased exposure to unimanual tasks, such as tool use and writing, over these years. In large part, the degree of hand preference may be a result of the amount of practice and experience a child has with certain motor skills.

### HAND PERFORMANCE

In an attempt to eliminate confounding variables inherent to self-report measures (e.g., subjective nature, difficulty understanding questions, and response items), researchers have implemented performance measures. Such measures are easy to administer, require relatively few instructions, and are less open to the subjective interpretation of the participant. Measurement tools of this nature assess the differences between the two hands on a given task to identify which hand demonstrates superior performance, and to quantify the difference in performance between the two hands (Peters and Durding, 1979; Annett, 1985). Performance differences that emerge are thought to reflect the degree or strength of hand preference (Provins and Magliaro, 1993), where most tasks assess manual strength, speed, accuracy, and precision. Numerous performance measures exist, including hand dynamometers to assess grip strength (e.g., Whipple, 1914; Daniels and Backman, 1993; Häger-Ross and Rösblad, 2002), dot-filling tasks (Tapley–Bryden dot-marking task; Tapley and Bryden, 1985), finger tapping tasks (Peters, 1980), peg-moving tasks (Annett Pegboard; Annett, 1970b, and Grooved Pegboard; Matthews and Klove, 1964), and manual aiming tasks (Roy and Elliott, 1986), among others. 

Early accounts of performance-based assessments of preference outline the use of hand dynamometer tests to assess strength differences between the two hands. For example, Whipple (1914), reporting on 6- to 18-year-olds stated that left hand strength averages 91–96% of right hand strength, observing that the strength of the preferred hand increases with age. Johnson (1925) examined 3- to 13-year-olds longitudinally with a dynamometer test. Over a year the percentage of left handers had decreased from 16 of 57 children to only one of 57 children. Daniels and Backman reported in their 1993 review that (1) grip strength increases with age; (2) male children are stronger than female children; and (3) right-handers are stronger with their preferred hand, whereas findings in left handers are inconsistent. More recent investigations (e.g., Häger-Ross and Rösblad, 2002; Molenaar et al., 2008; Koley and Melton, 2010) parallel these findings. 

Hand performance has also been examined with dot-filling (Tapley and Bryden, 1985) and finger-tapping (Peters, 1980) tasks (Singh et al., 2001), where performance is also observed to improve as a function of age. Carlier et al. (1993) performed Tapley and Bryden’s (1985) dot-filling task with left- and right-handed 7- to 15-year-olds. This task requires participants to place a dot in a circle following a pattern as quickly as possible. Dots are placed so each hand can work in its own region of hemispace. The preferred hand is used during the first and fourth trials, whereas the non-preferred hand is used in the second and third. Scores are averaged for both hands and a laterality quotient it computed to consider differences between preferred and non-preferred hand performance, and between left and right hand performance. Overall performance was similar for left and right handers, such that scores of both the preferred and non-preferred hand improved from the age of 7–14. That said, as a group, left handers were less lateralized than their right-handed counterparts. The degree of laterality was linked to age, where the direction of the effect was directly associated with the measurement. More specifically, when observing differences between the two hands, laterality increased with age (Carlier et al., 1993). 

In a subsequent study, Carlier et al. (1993) compared children’s performance on the dot-filling task with the finger-tapping test. Left- and right-handed 8- to 11-year-olds were instructed to tap a computer mouse with the index finger as quickly as possible from a “go” command until a “stop” command. Each trial time varied between 20 and 73 s. The dot-filling test was completed after the tapping test, either individually, or in the classroom as a group. As expected, older children performed faster and preferred hand scores were better than non-preferred hand scores. However, in comparison to Carlier et al. (1993) the difference between the two hands was not associated with age. The authors argued that the tapping task is not a complex skill that must be trained, whereas dot filling parallels writing skills, which is a learned trait. As such, results highlighted that task complexity plays a significant role in the size of the preferred-hand advantage. As such, the speed of dot-filling performance is better suited to differentiate the preferred hand, in comparison to variability of tapping. 

Taking into consideration the assessment of manual speed, research has also focused on peg-moving tasks, such as the Annett Pegboard (Annett, 1970b) and the Grooved Pegboard (Lafayette Instruments, Model # 3205; Matthews and Klove, 1964) to assess age-related changes in performance. The Annett pegboard measures manual speed by timing the movement of 10 dowelling pegs from a row of holes (one inch apart) on one board to an identical board located parallel, eight-inches away from the starting board. Participants move each peg individually, using only one hand, and a laterality quotient can be calculated to identify performance differences between the hands. Observing concordance between preference and performance measures, the Annett pegboard has been shown to identify 86.8% of right handers and 80.8% of left handers, regardless of age, as determined via the WHQ (Bryden et al., 2007a). Annett’s task may be better suited to assess age-related changes in performance, as it is easy to administer and seems to differentiate well between the hands. Similar to the Annett Pegboard, the Grooved Pegboard Test (Matthews and Klove, 1964) was designed to assess manual performance (Bryden et al., 2007a), however, the Grooved Pegboard requires a greater degree of manual precision to complete than the Annett pegboard. To complete this task, participants are traditionally asked to move 25 key-shaped pegs, individually, from a receptacle to an end position (place task). Modifications to standardized procedures, which require participants to remove the pegs and return to the receptacle (replace task), have been suggested to be a purer measure of motor speed (Bryden and Roy, 2005b) than the original placement task. 

Literature surrounding the development of unimanual performance for peg-moving tasks indicates that peg-moving time decreases as a function of age (Kilshaw and Annett, 1983; Curt et al., 1992; Singh et al., 2001; Annett, 2002; Dellatolas et al., 2003). In particular, Annett (2002) and Dellatolas et al. (2003) have noted an approximate 40% decrease in peg-moving time between the ages of 3 and 6, where variability in scores also decreased with age. Young children (3- to 6-year-olds) therefore perform significantly slower than older children and adults and with greater variability in their performance at 3-years-old in comparison to 6-years-old. This difference has been attributed to weaker lateralization evident in 3-year-olds which appears to strengthen with age. A longitudinal study (e.g., Fennell et al., 1983) provides support for this developmental aspect of hand preference. Reports of performance differences between the two hands also vary in the literature. Some studies indicate that asymmetry does not change as a function of age (e.g., Kilshaw and Annett, 1983; Annett, 2002; Curt et al., 1992; Dellatolas et al., 2003). As outlined by Annett, “differences are slightly larger in young than older children but this is a function of the rapid rates of growth in the early years” (p. 552). In comparison, others have noted significantly larger asymmetries for young children, in comparison to adults, who have the smallest performance differences between the hands. Likewise, the laterality quotient for children has been reported larger than what is observed for adults (Roy et al., 2003; Bryden and Roy, 2005a; Bryden et al., 2007a). Such effects have been found primarily for the Annett Pegboard. 

With respect to measures of hand performance with the Grooved Pegboard, researchers have determined that, for young children, performance differences between the hands are further exaggerated in tasks that require skill and precision. These differences disappear in 10- to 12-year-olds, as children’s performance becomes increasingly adult-like with age (Bryden and Roy, 2005a; Bryden et al., 2007a). With respect to adult performance, right handers have been observed to complete the task significantly faster with the right hand; however, performance differences between the hands are almost negligible, but still significant (Roy et al., 2003). 

The inconsistent findings in the literature highlight that, despite the obvious benefits of utilizing performance measures (e.g., fast and easy to administer), such measures have inherent limitations. It appears that tasks requiring precision aiming result in larger performance differences between the hands than less complex tasks (Carlier et al., 1993; Bryden and Roy, 2005a; Bryden et al., 2007a). Thus, each unimanual task likely measures only one aspect of manual performance abilities (e.g., speed or accuracy); however, there are likely several factors underlying performance differences between the hands, as handedness is a multidimensional trait.**Support for this suggestion comes from Corey et al. (2001), who measured hand preference of left- and right-handed adults using Briggs and Nebes’ and Oldfield’s handedness inventories, in conjunction with three of the aforementioned performance tasks (Grooved Pegboard, finger-tapping, and grip strength). Analysis revealed that use of one hand performance measure was not sufficient to classify an individual as left- or right-handed. However, using finger tapping and Grooved Pegboard scores together did enable correct classification of participants. Results of this study highlight that hand preference is a multidimensional trait; therefore, during assessment the numerous components of hand preference and performance must be considered (Corey et al., 2001). 

Summarizing, research using performance measures to assess hand preference has revealed that skilled unimanual performance increases as a function of age from early childhood to adulthood, where 3- to 6-year-olds show more variable and slower movements than children older than 6 and adult-like performance emerging between 10- to 12-years of age. The pattern of results suggests that practice, learning and experience play a role in refining the performance of both the preferred and non-preferred hands. The size of performance differences between the hands is a topic of debate, as some researchers have identified notable differences in younger children that decrease with age; whereas others have noted similar patterns of lateralization over the course of development. Such differences may in part be due to the performance tasks used in the studies, where learned tasks that require high levels of precision result in significant effects of age on the performance differences between the hands. In contrast, tasks that are less complex and not necessarily learned, such as finger tapping, may not show age-related changes in the size of the preferred-hand advantage. It is clear that investigators must choose carefully which performance task to utilize in research investigating manual performance differences in children. With respect to variations among handedness groups, the literature has shown that right handers demonstrate a greater preferred-hand advantage, where left handers display similar performance with both the preferred and non-preferred hand, as both children and adults. The performance of left handers on various unimanual tasks indicates these individuals are less lateralized in general than their right-handed counterparts. Because both hands of left handers are relatively equivalent in performance abilities, it may take developmentally longer for left handers to determine which hand is actually more efficient at performing particular tasks, hence explaining their variable performance on hand preference questionnaires.

### OBSERVATION OF HAND PREFERENCE

Given the problems of assessing hand preference in children using either hand preference questionnaires or performance tasks, Krombholz (1993, c.f., Kastner-Koller et al., 2007) therefore suggested measuring handedness through the use of video observations of children in their natural environment. Observational-based assessments of handedness have thus been implemented as an alternative measure of hand preference, where these measures are both appropriate and effective for use with children (e.g., Karapetsas and Vlachos, 1997). 

Hardyck et al. (1975) assessed handedness in a very large sample (*N* = 7688) of students in grades one to six. Left handers comprised 9.6% of the sample, with 10.5% of male children (*n* = 3960) and 8.7% of female children (*n* = 3728) being left-handed. Three behavioral tasks included handwriting, paper cutting, and picking up a paper tube to look through it (paper tube used to assess eye preference). Results revealed consistent hand preference for all the tasks, with no association with age. Because mixed handedness was observed so infrequently, the authors argued “to render unnecessary the categorization of mixed-handed” (Hardyck et al., 1975, p. 371). In a similar study, Nachshon et al. (1983) assessed laterality (hand, eye, and foot) preferences in a large sample of 7-year-old children. To measure hand preference, three colored pencils were placed at the midline and the child was asked to make an “X” on a piece of paper. If children used the same hand for all three pencils, the test was completed. If, however, children displayed inconsistent hand selection, the test was repeated twice more. A score less than four out of five was coded as variable. The authors observed greater than 80% of children were right-handed, where approximately 37% of children displayed consistent right and 3% displayed consistent left preference. 

Similar to Hardyck et al. (1975) and Nachshon et al. (1983), Coren et al. (1981) examined a battery of lateral preference tests in a group of 3- to 5-year-old preschool children and high school students (i.e., young adults). These performance tests assessed hand, foot, eye and ear preference, where specific hand performance tests included: (1) picking up a ball and throwing it to the experimenter; (2) touching the nose with a finger; (3) picking up a crayon and drawing a circle; (4) picking up a small ball with a spoon; and (5) cutting out a piece of paper with scissors. Based on performance of all items, each child was classified according to right-side, mixed or left-side preference. Analyzing hand preference dichotomously both pre-school and high school students demonstrated a strong right hand preference; however, when separating participants based on strength of handedness, a difference in age emerged. High school students were significantly more right-handed, thus highlighting that consistency of right hand use increases as a function of age. Using the same battery of tests, results were replicated by Longoni and Orsini (1988) in a group of 4- to 6-year-old preschool children. 

Rymar et al. (1984) also included a battery of performance items to assess developmental trends in hand preference. Six to 15-year-old elementary and junior high school students were observed performing various tasks: writing, throwing, using chopsticks, using scissors, drawing, hammering and using a spoon. Participants were classified according to left-, mixed-, or right-handed and data was analyzed according to age. Results highlighted fluctuating patterns of hand preference, such that students in the 6th grade of elementary school and 1st grade of junior high (11- to 13-year-olds) demonstrated the strongest hand preference. Similarly, Singh et al. (2001) observed the hand used to complete 10 simple tasks: writing, erasing, lighting a match, throwing a ball, hammering, using scissors, picking up small objects, brushing teeth, using knife, and combing hair. Hand preference responses were significantly different according to age, such that more 4- to 6-year-olds displayed weak hand preference tendencies in comparison to 7- to 11-year-olds. This shift from weak to strong hand preference was observed in both left- and right-handed children. 

More recently, Kastner-Koller et al. (2007) integrated 48 tasks (i.e., 16 tasks administered three times) of visuo-motor skill and general development into a treasure hunt. Using Steenhuis and Bryden’s (1989) ideas as a guide, movement components included (1) proximal movements, (2) distal movements, (3) grasping objects and (4) manipulating objects. Each component was performed in two stages: (1) precise skilled movements, and (2) fast, automatic movements. For example, precise proximal movements included throwing a ball and sweeping the floor, whereas an automatic proximal movement included pointing to a dot and waving. Pre-school and kindergarten children were observed completing the tasks by a trained examiner to assess hand preference, where use of a parent-report questionnaire and observations of hand preference for drawing enabled researchers to validate the task. Results of this investigation revealed that, in comparison to left-handed children, right handers were more lateralized in their direction of preference. That said, regardless of an overall left or right hand preference, children who demonstrated consistent preferred hand use had higher developmental scores (assessed with the Vienna Developmental Test; WET, Kastner-Koller and Deimann, 2002) than children who demonstrated hand-switching between tasks. Furthermore, right-handed and ambidextrous children were observed to have superior visuo-motor skills in comparison to their left-handed counterparts (Kastner-Koller et al., 2007). 

Where Kastner-Koller et al. (2007) opted for a treasure hunt to assess handedness in children, the WatHand Cabinet Test (WHCT^1^; previously referred to as the WatHand Box Test in Bryden et al., 2000a) is the observational measure of choice in our laboratory. Composed of a small, vertically oriented, two compartment cabinet with a door covering the top compartment, participants are asked to complete a complete a series of unimanual and bimanual tasks. These tasks include:

lifting the cabinet door a total of four times, using a toy hammer, placing rings on hooks, tossing a ball to a target, opening a lock with a key, using a screwdriver, pushing small buttons on a gadget, picking up a candy dispenser that was behind the cabinet door (Bryden et al., 2007b, p. 831).

Due to the number of tasks, several scores can be obtained from the WHCT, including a *skilled score*, a *consistency score*, a *bimanual score*, and finally, a *total score*. The *skilled score* is computed from seven tasks that require manual dexterity (use a toy hammer, place a washer on a hook, toss a ball to a target, open a lock with a key, use a screwdriver, push small buttons on a gadget, use a crayon). A laterality quotient [(*R *- L)/(*R *+ *L*) × 100] is computed, taking into consideration the number of tasks completed with the left and right hands. The *consistency score* is computed by averaging right-hand performance of the four unimanual door lift tasks (scored 0, 1, 2, 3, or 4 out of 4; Bryden et al., 2007b). In comparison, the *bimanual score* records the hand used to open the cabinet door in relation to the hand used to retrieve the candy dispenser. A score of 1 represents opposite hand use for opening the cabinet and reaching for the object, and a score of 2 represents use of the same hand for both elements. Finally, a laterality quotient [(*R *- *L*)/(*R* + *L*) × 100] is used to calculate a *total score* from unimanual tasks (Bryden et al., 2000a, 2007b). 

In order to assess the validity of the WHCT, Bryden et al. (2007b) completed the WHCT with 548, 3- to 24-year-olds (grouped 3- to 5-year-olds, 6-year-olds, 7-year-olds, 8-year-olds, 9-year-olds, 10-year-olds, 11-year-olds, 12- to 18-year-olds, and adults). Each participant also completed the Annett Pegboard and WHQ, to confirm hand preference, where both left and right handers were included in the study. Results revealed significant correlations between the WHQ (*r* = 0.795, *p* < 0.01) and the Annett Pegboard (*r* = 0.542, *p* < 0.01), therefore confirming the WHCT is a valid measure of hand preference to observe and quantify hand preference in individuals of all ages (Bryden et al., 2007b). Furthermore, the sub-scores of the WHCT provided a novel method of assessing different components of handedness in children. Notably, the skilled score assessed handedness similar to traditional assessments, which was likely due to overlap between tasks. It was thus suggested that the WHCT skilled score could be used individually to assess handedness (Bryden et al., 2007b). 

The aforementioned assessments of handedness in typical development with the WHCT (Bryden et al., 2000b) has demonstrated that young children (3- to 4-year-olds) are the least lateralized and consistent in comparison to older children (6- to 7-year-olds and 9- to 10-year-olds) and adults. It has thus been proposed that young children (3- to 5-year-olds) display weak hand preference tendencies until the age of 6, where hand preference is established and continues to strengthen as a function of age. In comparison to their right-handed counterparts, left-handed children show depressed scores when investigating consistency of hand preference. Some left-handed children appear to use their non-preferred at least half of the time. It is generally understood that left-handed children demonstrate significantly greater non-preferred hand use in comparison to their right-handed counterparts, who, independent of age, appear to use their preferred hand almost exclusively (Bryden et al., 2000b). Overall, the WHCT has been documented as the most accurate means of assessing hand preference in children due to minimal verbal requirements involved in the observational-based assessment (Bryden et al., 2007b). As such, Bryden et al. (2007b) suggested that the WHCT would be an excellent tool for use with special populations. In fact, the WHCT has been successfully used to assess hand preference in children with Autism Spectrum Disorders (e.g., Markoulakis et al., 2012). 

Summarizing then, observational assessments of hand preference have noted an increase in strength of hand preference with age. Such findings mirror those found using hand preference questionnaires noted earlier. In particular, young children, between the ages of 3- and 5-years-old, display weak, inconsistent hand preference tendencies. With age and maturation, hand preference becomes gradually more consistent, resulting in a shift from weak to stronger hand preference tendencies.

## DEVELOPMENT OF HANDEDNESS: NON-TRADITIONAL ASSESSMENTS

### MANUAL MIDLINE CROSSING

In addition to traditional measures, which quantify hand preference using preference, performance and observational assessments, researchers have also examined how hand preference influences hand selection in manual midline crossing. Manual midline crossing is observed when an individual reaches across the body midline into contralateral hemispace. To successfully execute such movement requires inhibition of the ipsilateral reach and, subsequent contralateral effort (Bishop, 1990). Failure to complete manual midline crossing has been well documented in the literature. For example, Head (1926) observed impairments first hand during the First World War, in which traumatized solders with aphasia were unable to complete contralateral movements. Researchers have since implemented manual midline crossing assessments to investigate developmental trends. 

Gordon (1923) developed a measure of children’s ability to cross the midline, observing an increase in ability with age. It is well understood that manual midline crossing is expected to emerge during infancy as a part of the typical progression of perceptual-motor development (Benton, 1959; Kephart, 1971). Bruner (1969) proposed the term “midline barrier” to describe infant’s early difficulties with contralateral movements, where research to date has outlined a specific developmental sequence underlying manual midline crossing. Infants’ initial reaches are primarily ipsilateral but progression to reaching for objects at the midline occurs quickly. Contralateral reaching begins to emerge between 18 and 20 weeks, reflecting the child’s exploration of their environment (White et al., 1964; Ball and Edgar, 1967; Wapner and Cirillo, 1968; Kephart, 1971; Greenman and Legg, 1976; Provine and Westerman, 1979; Liederman, 1983). It has been suggested that this period represents a shift from extracallosal to callosal control of interhemispheric communication. In particular Liederman (1983) noted that maturation of the corpus callosum is a required prerequisite for development of hand preference and bimanual coordination. Others have described that manual midline crossing is necessary for developing a skilled preferred hand (Provine and Westerman, 1979; Ayres, 1972, 1980). Manual midline crossing is well established in various tests by the age of 2 (Stilwell, 1987); however, reaching into contralateral space is a skill that gradually improves with age. Various methods of postural compensation are observed, as young children avoid contralateral reaching during visuomotor tasks (Roach and Kephart, 1966). It has been suggested that failure to cross the midline by the age of 3–4 may highlight, at an early state, problems with perceptual-motor development that will manifest later in life (Michell and Wood, 1999). 

Based on research in hemispatial neglect, line bisection tasks have been used to investigate age-related changes in manual midline crossing (Bradshaw et al., 1987; Bradshaw et al., 1988; Dellatolas et al., 1996; Van Vugt et al., 2000; Dobler et al., 2001; Hausmann et al., 2003). Young children avoid movements to contralateral space, using each hand in its own region of space and displaying patterns of “symmetrical neglect.” This behavior is typically observed from 4-years-old to 7- or 8-years-old (Bradshaw et al., 1987, 1988; Dobler et al., 2001), at which point adult-like patterns begin to emerge. Adults typically display “pseudoneglect” (Bowers and Heilman, 1980), where a line is transected to the left of center. Hausmann et al. (2003) noted that the shift from immature to mature control persists through the ages of 10- to 12, where numerous researchers have indicated developmental changes may parallel the transition from childhood to adolescence and subsequent maturation of the corpus callosum (Finlayson and Reitan, 1976; Dodds, 1978; O’Leary, 1980; Pujol et al., 1999; Giedd et al., 1996). 

In addition to line bisection tasks, researchers have also observed children’s willingness to cross the midline to reach contralaterally. Developmental trends have been noted in a variety of age groups using several different tasks. Schofield (1976) observed the tendency for 3- to 8-year-old children to make preferred hand contralateral responses in Head’s (1926) Hand, Eye and Ear Test. Children observed a female model touch her left or right ear or eye with the left or right hand and were asked to copy the movement. The number of preferred hand ipsilateral and contralateral responses was recorded. Younger children used each hand in its own region of hemispace and showed few if any contralateral reaches with the preferred hand. A gradual increase in preferred hand reaches across the midline was noted with age. However, the authors highlighted the lack of a “straight-forward developmental trend” (p. 576), as 4-year-olds were observed to cross the midline as often as 7- and 8-year-olds, and 6-year-olds made fewer movements across the midline than any other age group. The authors summarized that, although children were more likely to use the preferred hand, this was not always the case. Non-preferred hand responses were apparent, but less likely than preferred hand reaches, especially when reaching across the midline. 

Cermak et al. (1980), Cermak and Ayres (1984), Atwood and Cermak (1986) have used the Space Visualization Contralateral Use score (SVCU% = (number contralateral responses/total number contralateral + ipsilateral responses) × 100) of the Test of the Southern California Sensory Integration Tests to assess the percentage of contralateral preferred hand reaches to pick up a block. Cermak et al. (1980) observed that preferred hand reaches across the midline increased with age in left- and right-handed 4- to 9-year-olds. Nevertheless, because of the variability in each age group, no statistically significant differences emerged. As noted, skilled hand preference develops with age; therefore, it was likely that picking up a block did not possess the skill requirement to drive preferred hand selection. Cermak and Ayres (1984) questioned whether the SVCU score could be use to differentiate between typically developing children and those with learning disabilities. Guidelines discriminated between younger (5- to 7-year-olds) children, but no differences emerged at 8-years-old. In line with the current review, the authors suggested that assessing manual midline crossing in older children may require more stringent criteria. Atwood and Cermak (1986) thus investigated how block placement from the midline might influence developmental trends in contralateral reaching. Right-handed 5- and 7-year-olds completed the space visualization test, where blocks were placed 1.9, 7.62, and 15.24 cm apart. Results demonstrated that the distance between objects does play a significant role in manual midline crossing, as younger children displayed less contralateral reaches at far distances. 

Expanding on the knowledge gained from Cermak et al. (1980), which showed the frequency of midline crossing to gradually increase between ages 4 and 9, Stilwell (1987) completed “The Test of Manual Crossing” with 2- to 6-year-old children. Manual midline crossing was assessed with a center-hinged pegboard (see Stilwell, 1987, p. 786 for illustration). Participants were asked to place a peg in a designated hole, where the hand used for peg manipulation was recorded. Similar to Cermak’s group (Cermak et al., 1980; Cermak and Ayres, 1984; Atwood and Cermak, 1986), the percentage of reaches across the midline was observed to increase as a function of age, where the absence of contralateral responses was rare. As such, results demonstrated that by 2-years-old, manual midline crossing is very immature, but nevertheless well established. Interestingly, in contrast to Atwood and Cermak’s (1986) findings, the number of contralateral responses increased with increasing distance from the midline (i.e., from 5.08 to 15.24 cm). 

Similar pegboard apparatuses have been used to identify at which point individuals will make awkward unimanual movements with the preferred hand, and subsequently switch to the non-preferred hand to complete a task. Bryden et al. (1994) used a long pegboard (see Bryden et al., 1994 for illustration) and long dot-filling task. For the long pegboard task, two pegs (one small, one large) were placed in the first two holes (one small, one large) and participants were asked to “leapfrog” the pegs (i.e., large peg to next large hole to right, etc.) from one side of the pegboard to the other. Starting on the right side of the pegboard with the right-hand, participants were instructed to switch to their left hand when it felt appropriate to do so. The point at which participants switched hands was recorded. Similarly, with the long dot-filling task, a row of small circles was placed in front of participants, enabling participants to make a dot in each circle, starting with the right hand and switching to the left when comfortable. The point at which the participant switched hands was recorded. Using a laterality quotient to compute a magnitude of difference between the two hands, results successfully differentiated between left and right handers, where the long pegboard task proved to be a better assessment of handedness. Bryden et al. (1994) suggested the long pegboard provides a more objective assessment of handedness in comparison to a preference assessment. Furthermore, the authors stated the potential of such measure for use with young children and special populations; however, to date this has not been examined. 

Where the aforementioned studies investigated manual midline crossing in the horizontal plane, researchers have also examined reaching throughout regions of hemispace. In 1996, Bishop, Ross, Daniels, and Bright developed the Quantification of Hand Preference task, which assesses hand preference in three task conditions (card pointing, reaching and posting) with a manual midline-crossing element. For example, in the card-reaching task Bishop et al. (1996) placed three playing cards at 30-degree intervals in hemispace (three positions in contralateral space, one at midline and three in ipsilateral space), each at a distance of 40 cm from the midline. Participants were asked to pick up a card and place it in a box at the midline, where the hand used to pick up the card was recorded. Bishop et al. (1996) suggested that this particular type of reaching paradigm is better suited for quantifying differences between handedness groups in comparison to other, more traditional assessments (i.e., questionnaires and pegboards). Displaying high homogeneity and test-retest reliability (Doyen and Carlier, 2002), this test has been shown to discriminate between hand preference groups based on direction and degree (Bishop et al., 1996; Calvert, 1998; Doyen and Carlier, 2002). Additionally, Bishop (2005) has stated that the card-reaching task is sensitive to developmental processes. 

To assess performance on the card-reaching task from a developmental perspective, Carlier et al. (2006) completed the task with left- and right-handed children between the ages of 3 and 10. Adjustments were made to accommodate for children. For example, the number of cards retrieved per spatial position was doubled (i.e., originally 3, now 6) and an additional card was included so children did not realize they had reached for the last card. Additionally, to facilitate non-readers, numbered cards were replaced with familiar pictures. Finally the distance between the midline and the card was shortened from 40 to 25 cm. Manual midline crossings were recorded, where, similar to early studies with manual midline crossing (e.g., Cermak et al., 1980; Stilwell, 1987) a developmental trend was observed based on number of crossings and spatial position. Statistically significant differences emerged between the youngest (3- to 4-year-olds) and oldest (8- to 10-year-olds) children. Furthermore, the contralateral hand was used less often to reach to extreme regions of hemispace. These results were replicated by Doyen et al. (2008) with 6- to 24-year-olds, demonstrating that adolescents and adults cross into contralateral space less often than 7- to 12-year-olds, regardless of sex or hand preference. The authors stated that these findings suggest the development of manual preference is an influential factor in the decision to reach into contralateral space. With age and acquired motor skill, task complexity decreases, enabling participants to reach into ipsilateral space with either the preferred or non-preferred hand. This argument is supported by research from individuals with a variety of neurodevelopmental disorders including Developmental Coordination Disorder, Specific Language Impairment (Hill and Bishop, 1998), Down syndrome (Groen et al., 2008), and Trisomy 21/Williams Beuren syndrome (Gérard-Desplanches et al., 2006). 

As noted by Hill and Khanem (2009), the aforementioned studies, which assessed manual midline crossing with the Quantification of Hand Preference Task, were limited to the card-reaching task. All three components (pointing, reaching and posting) were thus completed with 4- to 11-year-old children (Hill and Khanem, 2009) to investigate how task constraints influence hand selection. As outlined previously, the reaching component required participants to pick up a specified card from hemispace and place it in a box at the midline. In comparison, the pointing task involved the least skill, requiring participants to point at a picture in hemispace; whereas, the posting task, which was the most challenging task, required participants to pick up a marble from the midline and post it into a cup with a small hole in its lid located in hemispace. Findings from other manual midline crossing tasks were replicated (e.g., Cermak et al., 1980; Stilwell, 1987; Carlier et al., 2006; Doyen et al., 2008). Task demands proved to influence hand preference when comparing reaching vs. posting and pointing vs. posting in contralateral space and at the midline. Distance from the midline also impacted the number of preferred hand reaches into contralateral space (Hill and Khanem, 2009). Younger children (ages 4–5) showed weaker hand preference being less likely to cross the midline in comparison to older children, and appeared to be more influenced by spatial position. 

The previously discussed studies measured what has traditionally been defined as limb preference, in that if an individual prefers one hand, then that limb would be selected to complete a variety of unimanual tasks. But what drives the choice of one limb for such goal-directed movements? Gabbard and Rabb (2000) argued that several process underlie the decision to select one limb over the other for reaching, including (a) limb dominance, as related to hand preference, and (b) attentional or spatial information associated with the demands of the task. More specifically, in most tasks, hand selection is driven by hand preference. However, once the preferred hand is biomechanically constrained by the degrees of freedom required to accomplish the task, and therefore unable to perform with the most efficient and comfortable response, the non-preferred hand is selected. In more simple terms, this behavior can be explained by hand selection according to object proximity. This is referred to as the *kinesthetic hypothesis* (Gabbard and Rabb, 2000; Gabbard and Helbig, 2004). Mark et al. (1997) have also suggested that postural dynamics guide hand selection and choice of reach. More specifically, people perceive the comfort of performing a reach with a single (arm only) or multiple (use of upper torso) degrees of freedom; therefore use the non-preferred hand in contralateral hemispace to avoid a multiple degrees of freedom reach. 

Another possible explanation is the *hemispheric bias hypothesis*, where each hand is used in its own region of hemispace, because performance (i.e., speed and accuracy) is greater in ipsilateral space (Bradshaw et al., 1990; Verfaellie and Heilman, 1990; Umilta and Nicoletti, 1992; Elliott et al., 1993; Hommel, 1993; Carnahan, 1998). To act in contralateral space requires interhemispheric communication, therefore results in decreased movement efficiency (e.g., Carson et al., 1992). Taking both hypotheses into consideration, it is suggested that hand selection may be initially driven by hand preference; however, information surrounding object location and task complexity may also be important (Bryden and Roy, 2006). 

To assess reaching with respect to the kinesthetic and hemispheric bias hypotheses Gabbard and colleagues (Gabbard et al., 1998, 2001) used a reach-to-grasp task with a block presented in nine regions of hemispace. Left- and right-handed 5- to 7-year-olds and adults were initially blindfolded with the hands placed at a rest position. Participants were instructed to remove the blindfold, return the hand to the rest position and keep the eyes closed until the “ok” signal was given. At that point, participants were instructed to pick up the cube and place it in a box at the midline. As expected, preferred hand use was observed more frequently in ipsilateral space. However, in contralateral space, most participants used the non-preferred hand. Overall, hand preference was observed to drive movements at the midline and in ipsilateral space; however, in contralateral space, kinesthetic and hemispheric biases led to non-preferred hand responses. Right handers were more consistent in hand selection tendencies than left handers, which indicates that hand preference is a stronger controlling feature when programming reach-to-grasp movements. 

In a related study, Leconte and Fagard (2006) had left- and right-handed 5- to 10-year-olds complete a task in which three identical objects (balls or dowels) were placed in left space, at the midline and in right space. Participants were instructed to grasp, grasp and relocate, or grasp the object and use it to pick up a sticker from the midline and relocate it. Results revealed greater preferred hand use in ipsilateral space and a shift to non-preferred hand use in contralateral space. Leconte and Fagard (2006) suggested that children “perceive the biomechanical constraints involved in the task and program the most efficient and comfortable response by using the hand closest to the object” (p. 91). The hemispheric bias hypothesis was also used to explain this behavior, such that use of the hand on the same side as the object was favored. Observing developmental trends, 5- to 6-year-olds used each hand in its corresponding region of space. Manual midline crossing was observed to increase with age, as observed previously (e.g., Cermak et al., 1980; Stilwell, 1987), where 10- to 12-year-olds were most likely to reach across the body with the preferred hand. Age-related changes are likely due to variable hand preference tendencies in early childhood, which become increasingly more consistent with age. 

To further delineate how individuals act in manual midline crossing, researchers have asked participants to manipulate the same object in varying contexts which alter the level of complexity. Bryden and Roy (2006; portions of study published in brief abstract/paper by Pryde et al., 2000) placed five toy objects at 45° in hemispace. Participants were asked to reach for an object in hemispace and complete a simple action (tossing the object) or a complex action (orienting and placing the object into a receptacle of the same size and shape). This enabled the researchers to examine the effect of task complexity, while recording the hand used to complete the task. Right-handed children (3- to 4-, 6- to 7-, and 9- to 10-year-olds) participated in this study, which observed use of the preferred hand through a greater range of hemispace than the non-preferred hand. Task complexity revealed no significant effects, which is likely due to the lack of sufficient degree of complexity. Similar to previous work, the authors noted a developmental trend in preferred hand use. Interestingly, 3- to 4-year-olds performed similar to adults, where preferred hand use decreased moving into left hemispace. In comparison, 6- to 10-year-old children used their preferred hand regardless of location in hemispace. This suggests that degree of hand preference in manual midline crossing is not consistent throughout development. The authors suggest that 3- to 4-year-olds use either hand at chance level, as they are exploring their environment. Children in the 6- to 10-year-old age range sacrifice cost-efficiency. Paralleling their stage of cognitive-motor development, they “tend to think in concrete, inflexible terms and are undergoing a period of motor skill refinement” (Pryde et al., 2000, p. 374). Finally, adults chose the most cost-effective movements, using their non-preferred hand in left space and preferred hand in right space (Bryden and Roy, 2006). 

As observed in the previous studies, object location and task characteristics influence hand selection over the course of development. This has led researchers to question how reaching for a tool (i.e., an object which affords a specific action; Gibson, 1979) as opposed to an object, influences selection of the preferred hand. In adults, the preferred hand is typically selected to use a tool. However, this is not necessarily true when simply picking up a tool (Bryden et al., 2003; Mamolo et al., 2004). Observing this scope of research from a developmental perspective, a breadth of literature has examined traditional reach-to-grasp tasks involving tools in infancy (e.g., McCarty et al., 1999; Claxton et al., 2009). However, there exists a dearth of investigations involving children. 

In a recent study Bryden et al. (2011) aimed to delineate how task complexity, object location and object type affects hand selection in children when considering a tool in comparison to an object with “no purpose” such as a dowel. Two hundred ninety-two right handers and 38 left handers (3- to 12- and 18- to 22-year-olds) were asked to pick-up and use one of five objects located in peripersonal space (five identical dowels and five tools: pencil, paintbrush, spoon, toothbrush, and a toy mallet or hammer), where the hand use to complete each element of the task was recorded. Results revealed that participants were more inclined to select their preferred hand when using a tool, as opposed to simply picking up the tool. Nevertheless, children did not differ in their hand selection for the different tasks when using the dowel, indicating the importance of tools and their saliency for action in hand selection. In line with previous findings (e.g., Calvert, 1998; Fagard, 1998; Gabbard and Helbig, 2004; Leconte and Fagard, 2006), children used their preferred hand most in ipsilateral space and at the midline, and tended to use their non-preferred hand in contralateral space. Furthermore, with age, the preferred hand was selected to a greater extent. Left-handed children showed an increased overall use of their preferred hand with age, whereas right-handed children used their preferred hand to the same extent across the ages. The only exception was 3- to 5-year-olds, who showed slightly depressed use of the preferred hand (Bryden et al., 2011). 

Overall, the research on manual midline crossing and hand selection suggests a similar pattern of development of handedness as seen from the more traditional methods of assessment. Children aged 3- to 5-years-old explore the environment and objects surrounding them, using the hand that is closest to the task to be performed in most cases. While the direction of hand preference may be established at this age, the skill level of the two hands has not yet been well differentiated. This exploration of the environment may be key in the child learning which hand is more effective and skilled at particular tasks. Children between the ages of approximately 6 and 10 years have learnt through experience which hand is more efficient and thus select this hand overwhelming, even in situations where it is not biomechanically efficient to do so. Between the ages of 10 and 12 years, an adult-like pattern of handedness emerges for all measures of handedness, as children learn to be less reliant on their preferred hand and the skill level of the non-preferred hand increases. The manner in which handedness emerges strongly indicates that experience, learning, and practice are key components in refining handedness and in particular an individual’s resulting degree of handedness.

## SUMMARY AND CONCLUSIONS

Identifying the origins of human hand preference and mapping the developmental trajectory has been at the root of neuropsychological and psychological research for centuries (e.g., Marshall and Magoun, 1998). Several researchers have suggested that hand preference may be associated with sensory-motor experience (e.g., Coryell and Michel, 1978; Nudo et al., 1996; Provins, 1997; Corbetta and Thelen, 2002) or environmental factors (e.g., Harkins and Michel, 1988; Harkins and Uzgiris, 1991; Provins, 1997); however, the belief that hand preference is rooted in genetics (e.g., Levy and Nagylaki, 1972; Annett, 1985; McManus, 1985; Corballis et al., 2012) has prevailed for numerous decades. 

The proportion of right and left handers in the human population has been described for approximately 5000 years (Coren and Porac, 1977). Annett (2002) and McManus (2002) have both stated that hand preference is a specifically human trait. Human hand preference appears to manifest itself very early in life (e.g., Corbetta et al., 2006; Michel et al., 2006) and run in families (Annett, 1972), which provides support for a genetic influence (e.g., Levy and Nagylaki, 1972; Annett, 1985; McManus, 1985; see Corballis et al., 2012 for a review of genetic and evolutionary bases). In fact, data shows that there is a greater likelihood of a child with one left-handed parent becoming left-handed, in comparison to a child with two right-handed parents (Annett, 1972; McManus and Bryden, 1992; McKeever, 2000). 

Although genetic accounts of hand preference seem plausible, they are limited in explaining individual development. For example, despite suggestions that hand preference emerges in infancy, variable hand-use strategies support the idea that hand preference is highly malleable (Corbetta et al., 2006) and different patterns of development exist (Michel et al., 2006). This variability is not limited to infancy. We have argued that there are three relatively distinct periods of refinement for handedness, and that experience, learning, and practice are key components at each of these stages. Young children (3- to 5-year-olds) typically demonstrate weak, inconsistent hand preference tendencies. This is particularly true for left-handed children. Young children are observed to use both hands to explore space. However, some object characteristics do influence hand selection. For example, hand selection preference is influenced when reaching to tools. Transitioning to older children in the 7- to 10-year-old range, there is an increase pattern of reliance on the preferred hand. Such strong, consistent hand preference ultimately drives performance differences between the two hands to increase, especially with respect to tasks requiring precision, as it is suggested the preferred hand is undergoing a period of motor skill refinement. Nonetheless, there is a relatively minute performance difference for speeded tasks. Children in this age range will select the preferred hand regardless of the task, object or position in space. This is thought to reflect children’s stage of cognitive motor development. 

Why are these variations in hand preference observed over the course of development? From a dynamic systems point of view, behavior emerges as one passes through life, “as the product of continuous intertwined reorganizations between multiple biological, environmental, and experiential factors that change and evolve as infants and children grow” (Corbetta et al., 2006). Handedness is thus sensitive to early sensorimotor experiences; however, after the foundation for basic motor skills is built, the motor system transitions to motor skill refinement (Corbetta and Thelen, 2002; Corbetta, 2005; Corbetta et al., 2006). This period of motor skill refinement is key to the development of handedness, and in particular the degree to which one prefers the dominant limb. Hand preference can be deemed adult-like when the reliance on the preferred hand drops (between the ages of 10 and 12 years), as performance differences between the two hands is small. This can be argued to be due to improvements in non-preferred hand performance due to additional experience and practice with manual skill. 

While past research has successfully utilized both preference and performance measures with children and adults to examine hand preference, we would argue that handedness assessed through observation of hand selection in reaching provides some of the richest data regarding the origins of hand preference and unimanual skill. We also suggest that while direction of hand preference, at least right preference, is established relatively early in life (and likely determined genetically), the degree of hand preference and size of the performance difference between the hands requires significant exposure to a range of motor tasks, both complex and simple, involving tools and other objects, to develop fully. Clearly, hand preference has arisen from the interaction of object, task, environmental and individual characteristics, and thus these variables need to be taken into consideration when exploring hand preference.

## Conflict of Interest Statement

The authors declare that the research was conducted in the absence of any commercial or financial relationships that could be construed as a potential conflict of interest.

## Footnotes

1 For instructions on the WatHand Cabinet Test please contact Dr. Pamela J. Bryden (pbryden@wlu.ca, 519-884-1979 ext. 4213), Department of Kinesiology and Physical Education, Wilfrid Laurier University, 75 University Avenue West, Waterloo, Ontario, Canada, N2L 3C5.
